# Health Consciousness and Its Effect on Perceived Knowledge, and Belief in the Purchase Intent of Liquid Milk: Consumer Insights from an Emerging Market

**DOI:** 10.3390/foods7090150

**Published:** 2018-09-07

**Authors:** Mohammed Ziaul Hoque, Md. Nurul Alam, Kulsuma Akter Nahid

**Affiliations:** 1 School of Business and Economics, UiT The Arctic University of Norway, Breivangviegn 23, 9010 Tromsø, Norway; 2 Coats Bangladesh Limited, Sagorika Road, Chittagong 4219, Bangladesh; mnaraju@rocketmail.com; 3 Department of Business Administration, International Islamic University of Chittagong (IIUC), Chittagong 4318, Bangladesh; shihabiha@gmail.com

**Keywords:** health consciousness, knowledge, belief, liquid milk, purchase intent, emerging market, Bangladesh

## Abstract

This study is based on the influence of consumers’ health consciousness (HC), perceived knowledge (PK) and beliefs affecting the attitude and purchase intent (PI) of the consumers. The outcome of this study is obtained through an exclusive survey conducted on a randomly selected sample of 712 households who purchase liquid milk (LM) in the cities of Dhaka and Chittagong in Bangladesh. A structured questionnaire is used to interview these participants to obtain data which are analysed employing descriptive statistics, Confirmatory Factor Analysis, and Structural Equation Modelling. The results of the analyses corroborate that consumers’ health consciousness has a positive impact on perceived knowledge, belief, and attitude, but not on purchase intent. In addition, belief affects both the attitude and PI positively. Although consumers’ perceived knowledge is too low to constitute their attitude towards LM, it has a positive, significant impact on the PI. The results also reveal that more than a third of the respondents consume LM several times per month, followed by more than a quarter of the sampled respondents who consume LM several times per week, and these consumption patterns have a positive and significant influence on the PI. Moreover, the monthly income of the family, age, and labelling preference are significantly correlated with PI.

## 1. Introduction

Global consumption and the market for functional foods pertain to, in large, consumers’ health consciousness [1] and their awareness, as well as knowledge [2]. As a result, studies that correlate between consumers’ knowledge in functional foods and their physical health are of great interest to researchers [3]. Research evidence indicates that Asia-Pacific is the fastest growing market and is expected to grow at a rate more than an eighth per year [4]. Similarly, the interest and demand for healthy food in the Bangladeshi market is projected to soar almost in the same manner [4]. Side by side, as disposable income [5] and organizations grow here [6], health consciousness among people and the demand for functional foods are growing up in this country [7]. Among functional foods, as the literature corroborates, milk is the most consumed and marketed processed dairy food product [8]. According to the statistics, per capita 13 L of milk per year, that is, more or less 35 mL of milk per day, is available to a person in Bangladesh [9], whereas at least 250 mL of milk per day is essential for every individual as per the standard of World Health Organization [10]. To maintain this standard, therefore, the consumption of dairy milk in Bangladesh should be multiplied seven times [11]. This large gap is the result of the inadequate supply of fresh liquid milk.

On the other hand, demand for liquid milk, according to the different reports in the literature, is affected negatively because the availability of pure and safe liquid milk (LM) from a reliable source is low in developing countries like Bangladesh [12]. Research shows that although households are less price sensitive in purchasing functional foods, in this case, quality and safe LM, they cannot avail themselves of their desired milk [13], since almost all of the milk samples, both raw and processed (both pasteurized and Ultra High Temperature (UHT)), are adulterated in many different ways, such as mixing water, cane sugar, powdered milk, starch, formalin or sodium bicarbonate [14], and are highly contaminated with fecal organisms [15]. Furthermore, since marketers have been supplying adulterated milk to meet the excess demand, regrettably, consumers are confused and they have less scope to believe that this LM is safe and fresh. Consequently, consumers prefer powdered milk to LM in order to avoid health risks [5]. Additionally, incidence of milk scandals in Bangladesh [16,17] and consumers’ low perceived value of belief have made them unhappy and, in most cases, have caused them to be more conscious about the quality of milk.

After being smeared by the effect of the supply-demand gap and food safety crisis, the consumers, vendors, policy makers, and other stakeholders currently accredit the importance of perceiving knowledge [18]. In case of purchasing LM, knowledge about the process and product is vital in explaining consumer behaviour [19]. Again, knowledge acquired on the products and values perceived by the consumers play a key role in determining the intent to purchase [20]. Literature revealed that health consciousness and information sources have an interacting effect on perceived knowledge in the use of nutrition labels [21]. In addition, the association between consumers’ health consciousness and knowledge is necessary to promote functional food consumption [2,3,22]. Then again, health consciousness is linked with subjects’ visits to health specialists [23], and perceived product benefits [24], indicating personal experiences. Again, personal experiences that subjects gather from different sources of information shape consumers’ belief [25], meaning a link between their health consciousness and belief. Furthermore, health consciousness prompts positive public service announcements for the use of nutrition labels [21]. Therefore, an urge from the demand side would be crucial to gauge the linkage between consumers’ Health Consciousness and Perceived Knowledge, and between Health Consciousness and Belief in predicting the behavioural intention of LM.

Consumers’ health consciousness and the necessity for functional foods are significantly correlated [1]. A recent study by Nahid [7] reported that health awareness is the only factor that influences the purchase intent of both processed and raw LM. Additionally, the ever-increasing awareness of gaining knowledge is mostly attributable to the health consciousness discerned by consumers [26]. As investigated by Ching-Hsu Huang [27], health consciousness does make changes to consumer preference, therefore influencing purchasing decisions of food. Numerous studies illustrate that health consciousness influences consumption [2,28,29], attitude and behavior [28,30], and word of mouth (WOM) [22]. However, the literature lacks information on how health consciousness influences consumers’ perceived knowledge and belief. Thus, the study aims to fill this knowledge gap.

In Bangladesh, around seventy percent people suffer from ‘Anaemia’ [31], forty percent suffer from chronic energy deficiency and majority of women suffer from osteoporosis [32]. As a fortified functional food, milk with vitamin D reduces risk of ‘osteomalacia’ and ‘osteoporosis’ [1], and milk has a positive influence on cognitive behaviour of humans due to its high level of vitamin D [33]. Additionally, the need for nutritional and quality food puts stress on vendors to supply unadulterated food [27], since to reach our genetic potential we have to drink milk regularly [34]. However, almost all sampled fresh milk traded on the Bangladeshi local market is adulterated [15]. One study has revealed that Asian consumers are health conscious when they reconsider drinking wine [35]. On the other hand, LM is considered to be the key functional dairy product for sale [36], thus, to know to what extent they are health conscious in drinking LM and how this consciousness is linked with their perceived knowledge and belief of LM could be interesting. However, in the context of Bangladesh, a promising market for dairy business development in south Asia, the influence of Health Consciousness, Perceived Knowledge and Belief in determining consumer attitude and purchase intent is yet to be discovered. As little is known, the later problems and knowledge gap motivate us to carry out this study. Thus, this study, in this context, aims to shed light helping design effective dairy policy and examining how consumers’ Health Consciousness is associated with their Perceived Knowledge and Belief, and what determining role Health Consciousness, Belief, and Perceived Knowledge play in forming attitude towards LM and in determining the purchase intent of LM using Structural Equation Modelling (SEM). Moreover, the association between these outlined factors were evaluated. 

The structure of the study is as follows. The literature review, hypotheses development, and the conceptual model are first demonstrated, followed by materials and methods. Then, the research results are discussed. Finally, the paper ends with concluding remarks, along with the limitations and directions for future research.

### 1.1. Literature Review and Hypotheses Development

People in developing countries currently consume an average of one-quarter of the milk and milk products per capita compared to the richer North, but this trend has been changing positively and rapidly. By 2020, developing countries are expected to consume 177 million metric tonnes more milk than they did in 1996–1998, and the success of the dairy industry presumably will depend on the protection of liquid milk [37]. Currently, the milk consumption rate has been increasing fast in Asia [38], where the 46 percent of total milk is consumed as LM [39]. In Bangladesh, as emerging tiger of Asia, the growth of demand for fresh milk is increasing, which is not commensurate with that of the production of milk [40]. Furthermore, the knowledge on the dairy field is scanty in this regard [40]. Therefore, if consumers’ Health Consciousness has an influence on knowledge and belief, and the Perceived Knowledge and Belief on Attitude and Purchase intent (PI) of LM, would be noteworthy for the development of functional foods and the dairy sector both in general, and in the emerging market. 

#### 1.1.1. Health Consciousness

Health consciousness refers to the extent to which an individual tends to undertake health actions [41]. Gould [42] framed health consciousness into four dimensions: greater concerns to health, caring about health, engaging in searching for health information, and valuing healthy conditions. To maintain a healthy lifestyle, a good dietary meal is of very importance, especially a functional meal. Numerous studies [43] have found that LM is a source of vitamins and nutrition that helps in maintaining good health. For instance, one glass (250 mL) of milk provides approximately 50% of the adult’s recommended dietary allowance [44]. As stated in the Gould’s [42] dimension, a health conscious individual should search for a source of healthy and fresh food. Therefore, we argue if a person is more health conscious, she/he is more likely to get involved in searching for fresh liquid milk that contains nutrition and vitamins, and in this way s/he might gather more knowledge.

**Hypothesis** **1a** **(H1a).** *More health conscious consumers will be more knowledgeable about LM than those who are relatively less health conscious.*

The literature shows that people who are health conscious tend to visit health specialists more often [23]. People who use media such as the internet, mass media, and interpersonal communication to gather health related information have proper health orientation, suggesting that the attitudes and behaviours [45] are linked with pursuing a healthy life [46]. Moreover, people with high concerns about health benefits consider LM as a source of diet and nutrition [24]. According to Fishbein and Ajzen [25], one of the bases that shape consumers’ belief is the personal experience they gather from different sources of information. Hence, it can be postulated that an individual’s knowledge [27,47] and thus, in turn, belief [45] regarding consuming fresh food, in this case LM, is linked with health consciousness that they perceive. Hence, the following hypothesis is posited:

**Hypothesis** **1b** **(H1b).** Consumers who are more health conscious will have a higher level of belief in LM than those who are less health conscious.

Health consciousness has been becoming a crucial factor while taking decision about consuming fresh food [27]. Again, consumers’ behaviour changes due to changes taking place in their individual life, such as health conscious preference, desire for healthy lifestyle, and so on. [27]. For this reason, providing information regarding nutritional value is important [48] and vendors are increasingly becoming aware, and providing quality food that has high nutritional value [27]. Hence, health consciousness influences attitude, and thus food purchasing decisions [49]. Therefore, the following hypotheses are formulated:

**Hypothesis** **1c** **(H1c).** Consumers’ health consciousness has a positive influence on their attitude towards LM.

**Hypothesis** **1d** **(H1d).** Health consciousness affects consumers’ intention to purchase LM positively.

#### 1.1.2. Perceived Knowledge

Consumer’s knowledge of food they purchase is crucial to explain consumer behaviour [19]. This knowledge is decomposed into two categories: subjective and objective knowledge. While subjective knowledge refers to an individual’s perception of the information about a certain product or attributes, objective knowledge refers to individual’s accurate information about the same. The more knowledge consumers perceive about the product, the less risk they are deemed to bear on [50]. Many researchers studied the interaction between knowledge and belief extensively [51,52]. Individuals’ knowledge about a certain event is shaped by the information they gain, while beliefs are based on knowledge an individual perceives [53]. The literature has also revealed that the product knowledge has impacts on consumers’ taste perceptions [54]. For the functional foods, consumers’ knowledge and their consumption frequency are significantly and positively correlated [29], and the consumers who have higher knowledge buy and consume more functional foods than others do [3]. In an emerging economy like Bangladesh, as a functional food, the extent to which consumers’ belief in LM is explainable by knowledge is yet to be measured. Thus, the following hypothesis is offered:

**Hypothesis** **2a** **(H2a).** Consumers with high level of knowledge will have high level of belief in LM.

In the case of purchasing fresh LM, knowledge about the process by which LM is collected, handled, and processed is vital to consider, since knowledge about unsafe food handling process could negatively impact the attitude towards food consuming decisions [55]. Consequently, in this study, consumers’ knowledge regarding LM is decomposed into two categories: product knowledge and procedural knowledge. Knowledge about the sources of collection (Raw or Processed etc.), quality, preservation status and so on is included in product knowledge [56]. Procedural knowledge entails different methods of processing or collection of LM to make it drinkable [56]. In addition, knowledge is expected to be highly correlated with consumption or expertise [57]. Considering the above discussion, the following hypotheses are designed:

**Hypothesis** **2b** **(H2b).** Consumers’ perceived knowledge has a positive impact on their attitude towards LM.

**Hypothesis** **2c** **(H2c).** Consumers’ perceived knowledge has a positive impact on the purchase intent of LM.

#### 1.1.3. Belief

That how consumers perceive a product or, how they react in the acceptance or rejection of a product is shaped by multidimensional factors [58]. These factors include both sensory attributes and prepossessed thoughts, such as belief about the products [59]. Studies suggest that consumers’ preferences regarding food choice can be greatly influenced by belief about the characteristics, as well as about the methods of processing [60]. As LM is a functional food and a convenient source of nutrition, people find it a good alternative. Hence, their belief can be shaped by the characteristics and attributes of LM. Additionally, as the LM has to go through the various collection processes, consumers’ perceptions can also be influenced by beliefs about the method of processing. 

Belief represents the information that a consumer possesses about an object and therefore links that object to some attributes [26]. Also, according to Smith, Walker, and Hamidova [61], belief can be represented as individual’s perception of the interaction between an object and the attributes related to it. Taylor [62] defined two types of belief namely personal belief and commonly held belief. The former is held in individual, solely inscribed in them and not shared with others. On the other hand, the latter is formed by few individuals, a small group, a community, a society, a culture or by most of humanity. Shared belief explains social action, which influences belief [62]. As a result, belief changes with the passage of time [62] and often is specific to groups or cultures. These findings give rise to the question of how beliefs are formed [63]. To address the factors constituting belief, Fishbein and Ajzen [25] proposed three bases for belief formation: descriptive belief, inferential belief and informational belief, suggesting that belief is formed through a very long and vigorous process. As a consequence, we can argue that belief is greatly comprehended by various aspects which, in turn, determine the consumers’ attitude, preference and purchase intent [60]. Hence, in the light of the above discussion, the following hypotheses are posited:

**Hypothesis** **3a** **(H3a).** Consumers’ belief has a positive influence in forming their attitude towards LM.

**Hypothesis** **3b** **(H3b).** Consumer’ belief has a positive influence in their purchase intent of LM.

#### 1.1.4. General Attitude and Purchase Intent

The Theory of Planned Behaviour (TPB) is used to elucidate a person’s intention to show a particular behaviour [64]. The stronger the intention, the higher the propensity to behave. The TPB assumes three independent factors, including the attitude toward the behaviour, the subjective norm and the perceived behavioural control (PBC) influencing the intention jointly [64]. According to the TPB, attitude serves as a key determinant of behavioural intentions. The more favourable the attitude of an individual towards the behaviour, the stronger his/her intention to perform the behaviour. Therefore, the following hypothesis is formulated:

**Hypothesis** **4** Attitude positively influences the purchase intent of LM.

**Figure 1:**
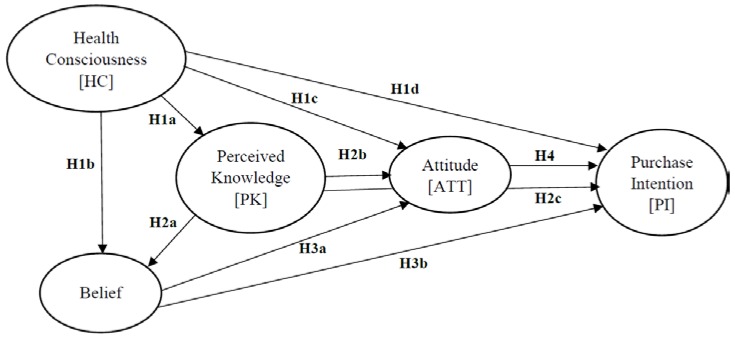
Conceptual model.

The study focuses on the role of Health Consciousness, Belief, and Perceived knowledge on consumers’ attitude towards LM and on their purchase intent. In doing so, the study develops a conceptual framework using structural equation modelling (Figure 1). 

#### 1.1.5. Mediating Effect of Health Consciousness

In general, one should discuss the direct, indirect, and total effects among latent variables in “causal effect modelling”, following the theory and the model [65]. An indirect effect implies the effect of an independent variable on a dependent variable through a mediating variable [66]. In food products, informed liking, such as consumers’ evaluation of packaging, brand, product variety, region, consumers’ blind liking, and so on, can mediate the relationship between cues and purchase intent [67]. As we are interested in testing the effect and relationship between consumers’ Health Consciousness and their Perceived Knowledge, and Health Consciousness and Belief, we assume the mediating effect can help in this regard. In doing so, the following four hypotheses have been posited.

Based on the H1a and H2b, Hypothesis 1e (H1e) is predicted:

**Hypothesis** **1e** **(H1e).** Consumers’ perceived knowledge regarding LM can mediate the relationship between Health Consciousness and Attitude.

Assuming a true relationship between the hypothesis H1a and hypothesis H2c, The second mediating hypothesis (H1f) is estimated:

**Hypothesis** **1f** **(H1f).** Consumers’ health consciousness has an indirect influence on the purchase intent through their perceived knowledge.

If the hypotheses, H1b and H3a are true, then we can posit the third mediating hypothesis:

**Hypothesis** **1g** **(H1g).** Health consciousness can contribute to forming consumers’ attitude towards LM via their perceived belief.

Finally, last mediating hypothesis, H1h has been formed based on the previously formulated hypotheses namely, H1b and H3b:

**Hypothesis** **1h** **(H1h).** Consumers’ health consciousness can influence the purchase intent positively through their perceived belief.

## 2. Materials and Methods

### 2.1. Participants and Procedure

The study was based on two major urban areas, Chittagong and Dhaka in Bangladesh. Dhaka is the capital city, while Chittagong is the chief port (business hub) and the commercial centre of Bangladesh. To test the hypotheses posted by the study, different methodological tools were used in the analysis. The data have been collected from the study areas by presenting a structured questionnaire. A sample of 712 households who prefer and consume liquid milk were selected randomly (364 respondents were from Dhaka and 348 were from Chittagong). In conducting the survey, the respondents were chosen in preference of convenience. The respondents were primary household shoppers defined as ‘the people who make purchasing decisions and regulate what the other members of the household eat’ [68], thus the credibility of the source was ensured. In addition, respondents older than the age of 20, and who are the buyer of LM, were chosen for the interview. The survey was carried out in an in-person survey, whereby the questionnaire was submitted to the consumers and they were asked to fill it in along with a face to face interview. Therefore, it was ensured that all questions be fulfilled, assuring the usability and completeness of the data. 

The data were collected by employing a structured questionnaire administered by enumerators in association with the researcher on various demographic and socio-economic characteristics of the consumers and their purchase intent and behaviour towards buying LM. The fieldwork was carried out during 1 April 2018 to 2 July 2018. Before the final version of the survey, a pre-test survey was conducted on 15 subjects in each city, in order to ensure that respondents understood the questions and no semantic and measurement problems exist. The interview on an average took 20 minutes per interviewee. The purpose of the study was stated in a cover letter and respondents were asked to answer a set of close-ended questions and statements designed to answer the questions. For strong inter-correlations, a sample size of 150 observations should be sufficient for reliable exploratory factor analysis (EFA) [69]. For confirmatory factor analysis (CFA), a minimum sample size of 100 is recommended [70]. Sekaran [71] considered the appropriate size of a sample to be between 30 and 500. Minimum requirements were, therefore, satisfied in the current study.

Descriptive analysis, EFA, CFA and SEM were used in the study. As EFA helps in summarizing the information received from a dataset, it is extremely useful to conduct EFA [72]. Here, EFA was used to determine an optimum number of dimensions, their mutual associations based on responses on particular items, and to form a pattern matrix. Based on the pattern matrix of EFA, CFA was used to justify the fitness of our model. SEM was used to measure the cause-and-effect relationship between the factors. For instance, to test H1a, ‘perceived knowledge’ was the dependent variable (DV) and ‘health consciousness’ was independent variable (IV). To test H1b, the ‘belief ‘was the DV and ‘health consciousness’ was the IV.

### 2.2. Questionnaire and Measure 

The questionnaire was divided into three sections, in which section one consisted of questions regarding the measurement of Perceived Knowledge, Health Consciousness, and Belief. Five questions were asked to measure Perceived Knowledge, five for Health Consciousness, and seven for measuring Belief. On the other side, section two included two parts: General Attitude, and Purchase intent. Six questions measured the general attitude, whereas purchase intent consisted of four questions. All questions were scaled in Likert five-point scale, from ‘Strongly disagree’ to ‘Strongly agree’. The questionnaire concluded with section three, wherein respondents’ demographics information including age, gender, occupation and so on were asked (see Section 3.1). 

The perceived knowledge scale has been constructed with the subjective judgement of respondents’ quality evaluation, level of knowledge about LM, expertise about the processing of LM, and information gathering [56,73]. For instance, participants were asked to answer to what extent they agreed with a statement like “Compared to an average person, I know a lot about LM”. The Health Consciousness scale considered the consciousness regarding respondents’ own health, family health, nutritional information, health related literature, and additive chemicals [27,48,74]. For example, participants were asked to answer to what extent they agreed with a statement like “I am self-conscious about my health”. The Belief scale has covered respondents’ beliefs about nutrition, taste, health, family recommendation, influence of WOM, influence of TV commercials, and doctors’ recommendation [75,76,77]. For instance, participants were asked to answer to what extent they agreed with a statement like “I believe that LM (processed or raw) is nutritious”. Purchase intent scale has considered five various types of intentions to purchase LM, when one buys milk next time, namely, she/he intends to buy, plans to buy, would like to buy, wants to buy, likely to buy LM [78]. For example, participants were asked to answer to what extent they agreed with a statement like “I intend to purchase LM next time I buy milk”. For the respondents’ rating, a five-point Likert scale, from “not at all” (1) to “totally agree” (5) was used. General attitude has been constructed with six Five-Point bi-polar scale items, such as bad to good, negative to positive, unfavourable to favourable, dull to exciting, terrible to great, and unsatisfied to satisfied [79,80]. 

However, the Exploratory Factor Analysis (EFA) has considered four questions for each of ‘attitude’ and ‘purchase intent’, three questions for ‘perceived knowledge’, two questions for ‘belief’, and two questions for ‘health consciousness. These factors all have eigenvalues greater than 1 (see Table 1), explaining 56.39% of total variance. Prior to EFA, two tests, namely Kaiser-Meyer-Olkin (KMO) and Bartlett's Test of Sphericity (BTS), should be verified for checking the factorability of data [81] and, evidently, the value of the KMO (in the first test) ranges from 0 to 1. However, for an appropriate analysis, the score should be at least of 0.60 and a BTS with the significant *p*-value (*p* < 0.05) [82]. The results were found to be significant (see Table 2). To test the reliability and internal consistency of the data, cronbach’s alpha for each of the constructs was also calculated (see Table 3). Composite reliability was tested (see Table 3). The discriminant validity test was conducted using correlation among constructs and comparing them with corresponding Average Variance Extracted (AVE) (see Table 4) to ensure that each construct is unique and that no multicollinearity problem exists in our data set. Finally, face validity confirmed the purpose for the usability of the constructs. 

## 3. Results

### 3.1. Demographic Profile of the Respondents

As the survey was conducted among the heads of the households, of the participants, the majority (63.1%) were male (Table 1), given Bangladeshi families are mostly headed by a male member. The rest 36.9% (263 participants) were female. The largest age group belonged to the 20–29 strata, with 270 members (37.9%), followed by the 40–49 age group that accounted for 171 members (24%). And the rest, 16.7%, 16.4%, 4.1% and 0.8% pertained to the 50–59, 30–39, 60–69 and above 70 age-groups respectively. To investigate whether the presence of children has any effect on the buying behaviour of LM, we queried for such information. A total of 451 respondents (63.3%) reported the presence of children in their family while the other 261 (36.7%) did not report having any children in their family. A majority of respondents (68.4%) reported having an education of above 12 years. This might ensure that, being educated, they reflect their true intention while responding. Most of the respondents consume LM several times per month, which accounted for 37.9% followed by 29.6% of population who consume LM several times per week. A significant 53.7% of the respondents opted for buying from local grocery shops and the other 17.1%, 14.7% and 14.5% from supermarket, Farm’s agent, and agro-farm respectively. 65.3% of the respondents (465 participants) reported that they read the label on the LM containing nutritional information, certifying authority and so on, while 34.7% of the respondents do not. For the certifying authority, the majority preferred the ‘national authority’ (32.6%), 106 respondents (14.9%) opted for ‘International authority’, a mere total of 14% opted for ‘local authority’ and ‘private authority’, while 160 responded (22.5%) that they have faith in ‘all equally’ and 110 respondents (15.4%) didn’t prefer any of the authorities. 

### 3.2. Measurement Model

Kaiser–Meyer–Olkin (KMO) and Bartlett’s Test of Sphericity, as suggested by Pallant [81], have been conducted prior to factor analysis. The KMO test achieved 0.723 (Table 2) which is well above the recommended 0.6 and ensured sample adequacy [82] and a significant *p* value is attained (*p* < 0.01) in the Bartlett’s Test of Sphericity, ensuring high likelihood of successful factorability of data [81].The measurement model demonstrated an excellent model appropriateness with the data having Chi square(*χ*^2^) = 105.52, Degree of Freedom (df) = 77, *p*-value = 0.017, Root mean square error approximation (RMSEA) = 0.023, Incremental fit index (IFI) = 0.993, Tucker-Lewis index (TLI) = 0.991, Comparative fit index (CFI) = 0.993, Goodness-of-fit index (GFI) = 0.981, Adjusted goodness-of-fit index (AGFI) = 0.970, and normed Chi square (*χ*^2^/df) = 1.37. It can be observed that model fit indices met the recommended threshold level as suggested by Hair et al. [83] and, hence, we were affirmed that the measurement components correspond to their underlying latent construct. 

Having an above recommended level result, EFA was run to test the convergent validity of the proposed constructs and to validate the factor loadings [84]. A total of 15 items (Table 3) have been derived with standardized regression weights (λ) ranging from 0.63 to 0.88, which is well above the recommended threshold level of 0.50 and all items were proposed to be significant on the corresponding latent constructs [84]. Furthermore, Cronbach’s alpha, which is considered to test the internal consistency, is calculated. For each of the four components, the minimum cut off value of greater than 0.6, as suggested by Hair, Black, Babin and Anderson [85], was achieved, though it is well recommended that Cronbach’s alpha be greater than 0.70. However, the reliability test reported a slightly lower value for the Belief construct, which is 0.57. We argue that it might have happened due to the nature of questions of the construct. All of the questions were connected to assessing belief, but they were different from each other in terms of nature and were not connected to a single dimension. On the other side, composite reliability (ρ), which is also used to test the internal consistency and the strength of the relations of the variables, was well above the recommended threshold level of 0.70 [83]. Further, each construct obtained an AVE value of above 0.50, although the AVE for four items of Belief and Perceived Knowledge is slightly below than 0.50 (see Table 3). However, these values are still acceptable as the composite reliability for the constructs is higher than/closer to minimum recommended level of 0.6 (see Table 3), thus ensuring adequate convergent validity of the constructs [86]. The reason behind this comparatively lower AVE could be the use of new measure applied in the field of LM assessing belief and perceived knowledge first time in the context of emerging economy like Bangladesh. In addition, we excluded those items among the constructs with poor factor loading and also testified to ensure that it does not create any major discriminant validity problems (Table 4).

Again, to test the discriminant validity, the AVE of each construct is compared with the respective correlations between the corresponding constructs, as suggested by Fornell and Larcker [86]. Here, in this case, as seen in Table 4, estimates for all variance extracted were greater than their respective squared correlation (*R*^2^), suggesting that each construct is unique in nature and that the dataset contains no multicollinearity problem. Furthermore, a value higher than 0.001 for the determinant of the correlation matrix of 0.001 in the model was found (determinant = 0.003), also showing no multicollinearity problems [87] (p. 445). Thus, discriminant validity is confirmed. To confirm the construct validity, it is necessary to assess the convergent validity, discriminant validity and face validity [88]. So far, convergent and discriminant validity are confirmed while face to face validity is a subjective judgement of whether measures of a certain construct “appears” to measure what it intends to measure. The theoretical support discussed in this section and data presented in the table confirms the face validity as well. Hence, we can conclude that there is a relatively good fit between our hypothesized model and observed data, and that the overall measurement model passes the validity of the analysis. 

### 3.3. Structural Model

#### 3.3.1. Assessment of Fitness for Structural Model

In order to gauge the fitness of the model, several goodness-of-fit test statistics were deployed [89]. To measure the impact of Health Consciousness, Perceived Knowledge, and Belief derived from it on consumers’ Attitude and Purchase Intent, the hypotheses presented in Figure 1 were tested. To test the hypotheses, the study develops a Structural Equation Model (SEM). SPSS and AMOS Graphics, 24.00 version (IBM, New York, NY, USA) were used for factor analysis and the path model analysis. 

An assessment of structural model (see Table 5) revealed that the data fit well with the proposed constructs. 

The RMSEA, GFI, AGFI, SRMR values were well above the recommended level, suggesting a good absolute fit index. The values for CFI, Non-Normed Fit Index (NNFI) and Normed *χ*^2^ also satisfied the recommended threshold level, ensuring both an incremental and parsimonious fit respectively. However, we found a chi-square value (*χ*^2^ = 139.881, df = 95) at *p* = 0.02. Since Chi-square has some limitations, including sampling sensitivity [94] and model misspecification norms [95], relative or normed chi-square (*χ*^2^/df) is suggested. Although no unanimous consent exists regarding an acceptable ratio for this measure, a ratio of below 3 to 5 is recommended [89].

#### 3.3.2. Results of Hypotheses Test and Correlations.

The primary purpose of this study was to examine the impact of various constructs, namely Belief, Health Consciousness and Perceived Knowledge, on the attitude and purchase intent of LM. Additionally, the association between them was also investigated by developing the hypothesized relationship in the light of previous theory and the literature. Table 6, below, shows the results to provide support for the acceptance and rejection of the hypotheses.

**Figure 2:**
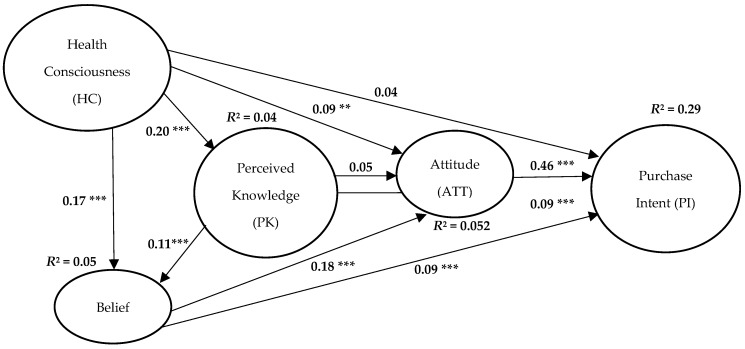
Results of hypotheses test on conceptual model. Note: *** significant at *p* < 0.01; ** significant at *p* < 0.05.

As seen in Table 6, and from Figure 2, a total of 10 hypotheses were tested, wherein eight hypotheses are found to be statistically significant. In H3a, Belief is found to significantly affect the Attitude towards LM (β = 0.18, Standard Error (SE) = 0.043, Critical Ratio (CR) = 4.84 and *p* < 0.001) as well as the Purchase Intent of LM in *H3b* (β = 0.09, SE = 0.042, CR = 2.76 and *p* < 0.01). Hypotheses H2a (β = 0.11, SE = 0.032, CR = 2.99 and *p* < 0.01), and H2c (β = 0.09, SE = 0.036, CR = 2.56 and *p* < 0.01) are also accepted, signifying that Belief and Purchase Intent are also positively affected by Perceived Knowledge they behold regarding LM. However, although, in H2b, Perceived Knowledge positively influences the attitude of the people regarding LM choice, the result is not statistically significant (β = 0.05, SE = 0.038, CR = 1.26 and *p* > 0.10). As predicted, in H1a, Health Consciousness positively influences Perceived Knowledge (β = 0.20, SE = 0.039, CR = 5.5 and *p* < 0.001), in H1b Belief (β = 0.17, SE = 0.035, CR = 4.56 and *p* < 0.001) and in H1c Attitude (β = 0.09, SE = 0.041, CR = 2.26 and *p* < 0.01). However, Health Consciousness cannot influence the Purchase Intent in H1d (β = 0.04, SE = 0.039, CR = 1.16 and *p* = 0.246). As predicted in H4, Attitude significantly influences the Purchase Intent (β = 0.46, SE = 0.035, CR = 14.13 and *p* < 0.001). 

In addition to explaining the impact of indigenous variables, this study attempted to explore the impact of moderating, mediating and demographic factors on Purchase Intent. A statistically significant positive effect of overall milk consumption on the Purchase Intent of milk has been found (β = 0.089, SE = 0.024, CR = 2.67 and *p* < 0.01). Significant negative correlation has been found between Income and Purchase Intent (*R*^2^ = −0.080, *p* < 0.1), between Age and Purchase Intent (*R*^2^ = −0.071, *p* < 0.1), and between Labelling and Purchase Intent (*R*^2^ = 0.075, *p* < 0.1). This indicates that people with higher income tend to have lower intention in buying LM. Additionally, people of a higher age do not prefer to consume much LM compared to people of a younger age. Most importantly, people who read the labelling of certification tend to consume more than those who do not read the labels and certification.

#### 3.3.3. Mediating Effect

This study also attempted to explore the mediating effect, if any, among the components. In this test, the influence of a third variable was investigated; whether it had any mediating role in explaining the hypothesized relationship between the given independent variable (IV) and dependent variable (DV) [96]. In addition, the values for two other tests, namely the Aroian test [97] and the Goodman test [98], were also provided in Table 7. As seen in the table, the results provide full support for the hypotheses H1f, H1g and H1h. However, the results do not support mediation for the hypothesis H1e, hence is rejected. The results indicate that while Health Consciousness regarding LM predicts Purchase Intent of consumers (H1d), the relationship can also be explained via Perceived Knowledge in H1f (*t* = 2.34, *p* < 0.05). Again, the effect of Health Consciousness on Attitude is significantly mediated by Belief, as in hypothesis 1g (H1g) (*t* = 3.31, *p* < 0.00), measuring the extent to which Attitude (DV) changes when Health Consciousness (IV) is held fixed and Belief (mediating variable) changes by the amount it would have changed had the Health Consciousness (IV) increased by one unit [99]. Similarly, H1h reveals that the effect of Health Consciousness on Purchase Intent is significantly mediated by Belief (*t* = 2.37, *p* < 0.05). 

## 4. Discussion

The study aimed to investigate the role of consumers’ health consciousness on perceived knowledge and belief in the first place. Then, the influence of health consciousness, perceived knowledge, and belief in forming attitude and purchase intent of liquid milk has been examined. In doing so, a structured questionnaire was developed using various scales supported by previous literature, and was carried out in the two major cities of Bangladesh. To address the research objectives, firstly, a total of ten hypotheses were formulated and tested using Structural Equation Modelling. In addition, a mediating role played by the constructs has also been investigated to see which variables, if any, play an indirect effect to explain the purchase intent of liquid milk. The data, measurement model and structural model represented adequate fit, and the reliability was also significant for various constructs.

The hypothesis H1a supports the argument that health consciousness significantly increases the perceived knowledge. Again, in H1b health consciousness positively signifies Belief. Jayanti & Burns [100] suggest that consumers with greater health knowledge displayed significant preventative health behavior, meaning that the purchase intent of liquid milk as a functional food was predicted by health consciousness. However, our findings contradicted theirs; after examining this issue in H1d we found that health consciousness does not have any significant effect on the purchase intent of liquid milk, although the impact of health consciousness is significantly positive on Attitude toward liquid milk (H1c). We argue that the milk adulteration incidence in recent decades in Bangladesh might have raised a negative concern among the consumers about the attributes of LM, and have a negative effect on the purchase intent. 

Belief formation is an extensive process [25] which is largely influenced by the perceived knowledge of a consumer [53]. Hypothesis H2a supports that perceived knowledge significantly and positively influences the belief of the consumer, suggesting that consumers who have stronger belief in liquid milk tend to bear more knowledge than those who have lower belief. This might be caused by the search for information relating to health and nutritional benefits of liquid milk, because of its features as a functional product, which is defined as foods sold for health benefits and is characterized by functional ingredients deemed to help prevent various diseases, and dietary supplements [101]. This finding is accentuated by testing hypothesis H2b and H2c, wherein H2b posits that perceived knowledge has no bearing on attitude toward liquid milk and H2c reports perceived knowledge has significant impact on the purchase intent of liquid milk. In line with these sorts of findings, Cazacu [102] reported that in Greece the perceived knowledge plays a significant role in purchase intent of dairy functional foods.

Accordingly, H3a supports that consumers’ belief plays a significant positive role in determining attitude towards liquid milk. In addition, H3b posits that the purchase intent of liquid milk is significantly and positively influenced by the belief. In line with this, the literature reports that an individual’s response toward a particular idea or object, predisposed by an organization of belief, can be defined as attitude [103], which, in turn, is hypothesized reflect on his behavior [104]. Again, a study conducted by Lino et al. [105] reported that underlying belief about dietary supplements significantly predicted the intention towards dietary supplement use. Moreover, belief regarding nutritional value, taste, freshness and appearance influenced organic food consumers’ attitudes and preferences [106]. Finally, H4 reports that the attitude is a good predictor of purchase intent, which also corroborates the basic attitudinal research that attitude is the precedent of purchase intent [107,108].

However, to test the mediating relationship between health consciousness and attitude via perceived knowledge, hypothesis H1e was investigated, and the result shows that perceived knowledge does not mediate the effect of health consciousness and attitude. Although health consciousness has a significant effect on perceived knowledge, for the low level of knowledge about liquid milk, perceived knowledge cannot mediate the relationship between health consciousness and attitude. Again, in hypothesis H1f, perceived knowledge can mediate the relationship between health consciousness and purchase intent. However, the results revealed that perceived knowledge cannot mediate the relationship between health consciousness and attitude, and consumers’ perceived knowledge is average or low. Therefore, an indirect effect of health consciousness on purchase intent through perceived knowledge indicates that they are purchasing liquid milk against their willingness to buy. Evidence shows that the sensory perceptions of liquid milk can mediate the relationship between perceived knowledge and consumers’ purchase intent [109]. Thus, perceived barriers influence consumers’ attitude negatively [110]. The hypothesis H1g supports the claim that health consciousness has an indirect effect on attitude through belief as perceived knowledge has a significant effect on belief. However, the same relationship is not true via perceived knowledge, indicating that consumers’ level of belief toward liquid milk is higher than their perceived knowledge. Because, the availability of information on personal attitude and belief and the cultural context in which this information is derived can also facilitate comprehension of the relative importance of factors that influence food choice [111]. Thus, for hypothesis H1h, the results support the hypothesis: belief can mediate the relationship between health consciousness and purchase intent.

## 5. Conclusions 

The main theoretical contribution of the paper is to conceptualize and model the factors influencing consumers’ purchase intent for liquid milk based on a field survey, including Health Consciousness, Perceived Knowledge and Belief. To the best of our knowledge, the study is a pioneer in the field of consumer behavior analysis in that it includes these three factors exclusively for the first time to explain their role in forming attitude and purchase intent, to get a close insight into consumer perceptions of liquid milk as a functional food and to help make an effective dairy policy. Our study reveals that health-conscious people do bear a positive attitude toward liquid milk, but their health consciousness does not necessarily influence their purchase intent. Consumers are becoming more educated, health conscious, and thus have begun to consider food attributes more carefully when choosing food items [112]. Again, health consciousness influences perceived knowledge and belief positively. Furthermore, perceived knowledge influences consumers’ belief positively, meaning that consumers’ health consciousness and their belief towards liquid milk have been developed based on their perceived knowledge. Thus, the marketers should focus on consumers’ knowledge about LM. Because the perceived knowledge of milk, like ‘taking responsibility for one’s health’, is the key to liquid milk commercialization [109].

The results of the study demonstrate that the influence of belief on both attitude and purchase intent is significantly positive. Existing literature shows that belief is formed through a long comprehensive process, such as in the form of direct observation, inferences, or gathering information [25,61]. Therefore, stakeholders should take initiatives to disseminate all required information, specifically the nutritional facts [109] that can help to increase the level of belief regarding their marketed liquid milk, since consumers perceive that behavioural belief can help producers, marketers and government in formulating effective dairy policies [7]. Again, the study by Boniface and Umberger [113] found that over hedonist consumers consider information regarding health, product, process, and convenience when they form certain perceptions regarding dairy product quality. That is the reason why manufacturers and marketers should know consumers’ perception toward the quality of a dairy product [114] because consumers who perceive dairy products as a nice source of nutrients consume more dairy products than do others.

From managerial perspective, this study substantiates the fact that the stakeholders, including marketers, government and different community actors, are far from educating the consumers about liquid milk, suggesting that an efficacious public awareness strategy should be increased through effective means of communication. This finding would have several implications so far. For instance, a more engaging consumer knowledge enhancement plan should be framed with ensuring the cognitive evaluation process so that their sentimental mentality of fear and emotional responses arising from the food safety concern could be diverted towards a more discreet purchasing preference decision [19]. 

The outcomes also report that health consciousness and belief influence consumer’s attitude towards liquid milk positively, but perceived knowledge does not have any statistically significant impact. However, perceived knowledge can affect purchase intent directly, meaning that consumers have little information regarding liquid milk that cannot help in building a significant feeling towards liquid milk. Evidence shows that in the Bangladeshi market, consumers are not satisfied with the quality of liquid milk [7,109]. In general, they consider the liquid milk to be a hedonic product [109] and recommend it for their children because they prefer to consume functional foods in the natural form [115]. Consumers' perceived value of naturalness and freshness of raw liquid milk are high [109] and we argue that consumers perceived value of naturalness of liquid milk is higher than other types of milk, such as powdered milk. As their perceived knowledge is poor and they do not have a better alternative in the market within a liquid category, until now consumers’ perceived knowledge influenced their purchase behavior of liquid milk. 

In the light of the following limitations, the future research scope can be identified. First, this study takes into account one dimension of health perception, namely physical health consciousness. Other dimensions such as mental, social, emotional, and spiritual health have not been considered. Future studies can examine these factors. In addition to this, the other important variables, such as the effect of emotion, specific belief, perceived risks, trust, and so on, can be used as an explanatory variable. For the convenience of the study, we administered a random sampling procedure; in the further research, a large sample with a greater range of cities should be used to ensure the efficacy of the model and to reduce the risk of spurious findings linking attitude and actual behaviour. In this study, we took into account the effect of subjective knowledge on attitude and purchase intent, but the magnitude of this discrepancy, namely, over and underestimation of knowledge, was not considered. However, future researchers should concentrate on their cautiousness about their insufficient knowledge regarding liquid milk by measuring and evaluating the subjective knowledge and objective knowledge separately. Hence, a further examination can consider this issue. Lastly, a cross-culture study should be undertaken to gain a deeper insight of the region and to design an effective strategy with a broader perspective.

## Figures and Tables

**Table 1:** Demographic profile of the respondents.

Categories	Sub-Categories	Frequency	Valid	Mean	SD
Gender (coded as M = 1, F = 2)	Male	449	712	1.37	0.483
	Female	263			
Age (1 = ‘Between 20–29’… 6 = ‘Above 70’)	Between 20–29	270	712	2.35	1.292
	Between 30–39	117			
	Between 40–49	171			
	Between 50–59	119			
	Between 60–70	29			
	Above 70	6			
Income * (1 = ‘<30,000’… 5 = ‘above 90,000’)	<30,000	372	712	1.80	1.061
	30,000–50,000	193			
	50,000–70,000	86			
	70,000–90,000	36			
	Above 90,000	25			
Children (under age of 16) (1 = Yes, 2 = No)	Yes	451	712	1.37	0.482
	No	261			
Education (1 = ‘0–5’… 3 = ‘>12’)	0–5	89	712	2.56	0.705
	5–12	136			
	>12	487			
Family Member (1 = ‘1–5’… 3 = ‘above 10’)	1–5	504	712	5.19	2.318
	6–10	194			
	Above 10	14			
Consumption (1 = ‘Several-time/month’, … 5 = ’Several-time/daily’)	Several-time/month	270	712	2.32	1.218
	1/month	101			
	Several-times/week	211			
	Daily	105			
	Several/daily	25			
Do you do most of the food shopping for your family? (1 = ‘Yes’, 2 = ’No’)	Yes	503	712	1.29	0.705
	No	209			
I bought milk (at least one time) in the last 4 weeks. (1 = Yes, 2 = No)	Yes	586	712	1.18	0.382
	No	126			
I buy milk from (1 = ‘Farm’, … 4 = ‘Retail grocery shop’)	Farm	103	712	3.08	1.133
	Super market	122			
	Farm’s agent	105			
	Retail grocery shop	382			
I read labelling on LM while purchasing. (1 = ‘Yes’, 2 = ‘No’)	Yes	465	712	1.35	0.476
	No	247			
Certification (1 = ‘A local authority’, … 6 = ‘Not any at all’)	A local authority	53	712	3.84	1.450
	A private authority	51			
	A national authority	232			
	An international authority	106			
	All equally	160			
	Not any at all	110			

Note: * 1 USD = BDT 82 (approximately); SD = Standard Deviation; M = Male, F = Female.

**Table 2:** Kaiser–Meyer–Olkin (KMO) and bartlett’s test of sphericity.

KMO and Bartlett’s Test		Score
Kaiser-Meyer-Olkin Measure of Sampling Adequacy		0.855
Bartlett’s Test of Sphericity	Approx. Chi-Square	4217.8
	df	105
	Sig.	0.000

Note: df = Degree of Freedom; Sig. = Significant.

**Table 3:** Measurement model.

Constructs and Items	λ	α	ρ	Eigenvalues	AVE
Purchase Intent		0.88	0.87	4.96	0.64
I want to buy LM next time I buy Milk	0.883				0.72
I would like to buy LM next time I buy Milk	0.840				0.71
How likely is it that you will buy LM, next time you buy milk	0.739				0.58
I am planning to buy LM next time I buy Milk	0.725				0.61
Attitude		0.88	0.87	2.01	0.63
Unfavorable to Favorable	0.914				0.66
Bad to Good	0.787				0.71
Terrible to Great	0.737				0.66
Negative to Positive	0.728				0.60
Perceived Knowledge		0.66	0.71	1.39	0.44
I have in depth knowledge to evaluate LM	0.691				0.50
Compared to an average person, I know a lot about LM	0.676				0.44
friends consider me as an expert in the domain of LM	0.640				0.41
Health Consciousness		0.70	0.68	1.27	0.52
I am self-conscious about my health	0.790				0.58
I am self-conscious about my family health	0.649				0.50
Belief		0.57	0.57	1.10	0.40
My doctor believes that I should take LM	0.638				0.43
I believe that LM is very significant to have a good health	0.630				0.37
Total Variance Explained	56.39%				

**Note:** λ—Standardized regression weights; α—Cronbach’s alpha; ρ—Composite Reliability; AVE: Average Variance Extracted; LM: liquid milk.

**Table 4:** Descriptive statistics and correlations among constructs.

Items	Mean	SD	Purchase Intent	Attitude	Perceived Knowledge	Health Consciousness	Belief
Purchase Intent *	3.87	0.885	(0.64)	0.372	0.052	0.038	0.144
Attitude *	3.55	0.808	0.610	(0.63)	0.020	0.041	0.118
Perceived Knowledge *	3.22	0.802	0.228	0.142	(0.44)	0.088	0.067
Health Consciousness *	4.06	0.742	0.194	0.202	0.296	(0.52)	0.128
Belief *	4.11	0.697	0.380	0.344	0.259	0.358	(0.40)
Determinant of Correlation Matrix 0.003 > 0.001 **							

* measured in Likert 5-point scale. ** Determinant of correlation matrix of 15 items of 5 constructs of the model. Note: The diagonal values in the parentheses represent AVE. The lower diagonal value represent correlation between the constructs whereas the upper diagonal values represent squared correlation between the constructs.

**Table 5:** Goodness of fit indices.

Category	Indices	Recommended Least Value	Attained Value
Absolute Fit	RMSEA	<0.08 [85,90]	0.026
	GFI	>0.90 [91,92,93]	0.98
	*χ* ^2^	*p* > 0.05 [93]	*p* = 0.02
	AGFI	>0.90 [93]	0.96
	SRMR	<0.05 [93]	0.03
Incremental Fit	CFI	>0.90 [93]	0.95
	IFI	>0.90 [93]	0.95
	NNFI (TLI)	>0.90 [93]	0.92
Parsimonious Fit	*χ*^2^/df (normed *χ*^2^)	<3–5 [93]	1.47

Note: RMSEA = root mean square error approximation; GFI = goodness-of-fit index; AGFI = adjusted goodness-of-fit index; SRMR = Standardised Root Mean Squared Residual; CFI = comparative fit index. NNFI = non-normed fit index; TLI = Tucker-Lewis index; IFI = Incremental fit index.

**Table 6:** Results of structural equation modeling: standardized path estimates.

Structural Path	Hypotheses	Standardized Path Co-Efficient (β)	SE	CR	*p*-Value
Belief → Attitude	H3a	0.18	0.043	4.84	0.000 ***
Belief → Purchase Intent	H3b	0.09	0.042	2.76	0.006 ***
Perceived Knowledge → Belief	H2a	0.11	0.032	2.99	0.003 ***
Perceived Knowledge → Attitude	H2b	0.05	0.038	1.26	0.207
Perceived Knowledge → Purchase Intent	H2c	0.09	0.036	2.56	0.009 ***
Health Consciousness → Perceived Knowledge	H1a	0.20	0.039	5.55	0.000***
Health Consciousness → Belief	H1b	0.17	0.035	4.56	0.000 ***
Health Consciousness → Attitude	H1c	0.09	0.041	2.26	0.024 **
Health Consciousness → Purchase Intent	H1d	0.04	0.039	1.16	0.246
Attitude → Purchase Intent	H4	0.46	0.035	14.13	0.000 ***
Correlations		**Pearson Correlation**			
Income and Purchase Intent		−0.080			0.035 *
Education and Purchase Intent		0.020			0.594
Age and Purchase Intent		−0.071			0.057 *
Gender and Purchase Intent		−0.039			0.304
Presence of Children and Purchase Intent		0.026			0.482
Labeling and Purchase Intent		−0.075			0.046 *

Note: *** significant at *p* < 0.01; ** significant at *p* < 0.05; * significant at *p* < 0.10. SE = Standard Error; CR = Critical Ratio.

**Table 7:** Results of mediating effect.

Mediating Path	Hypothesis	Test	Test Statistics	Standard Error	*p*-Value
HC → PK → ATT	H1e	Sobel Test	1.231	0.008	0.218
		Aroian Test	1.213	0.008	0.225
		Goodman Test	1.251	0.008	0.210
HC → PK → PI	H1f **	Sobel Test	2.343	0.008	0.019
		Aroian Test	2.312	0.008	0.020
		Goodman Test	2.374	0.008	0.017
HC → Belief → ATT	H1g ***	Sobel Test	3.315	0.010	0.000
		Aroian Test	3.278	0.010	0.001
		Goodman Test	3.353	0.010	0.000
HC → Belief → PI	H1h **	Sobel Test	2.37	0.007	0.019
		Aroian Test	2.32	0.007	0.017
		Goodman Test	2.41	0.007	0.015

Note: ** significant at *p* < 0.05; *** significant at *p* < 0.01; ATT = Attitude; PI = Purchase Intent: PK = Perceived Knowledge; HC = Health Consciousness.
